# Resonance-Based Sensing of Magnetic Nanoparticles Using Microfluidic Devices with Ferromagnetic Antidot Nanostructures

**DOI:** 10.3390/nano14010019

**Published:** 2023-12-20

**Authors:** Reyne Dowling, Ryszard Narkowicz, Kilian Lenz, Antje Oelschlägel, Jürgen Lindner, Mikhail Kostylev

**Affiliations:** 1 Department of Physics, The University of Western Australia, Crawley, WA 6009, Australia; reyne.dowling@research.uwa.edu.au; 2 Institute for Ion Beam Physics and Materials Research, Helmholtz-Zentrum Dresden-Rossendorf, 01328 Dresden, Germany; r.narkovic@hzdr.de (R.N.); k.lenz@hzdr.de (K.L.); j.lindner@hzdr.de (J.L.)

**Keywords:** ferromagnetic materials, microfluidics, soft lithography, superparamagnetic iron-oxide nanoparticles

## Abstract

We demonstrated resonance-based detection of magnetic nanoparticles employing novel designs based upon planar (on-chip) microresonators that may serve as alternatives to conventional magnetoresistive magnetic nanoparticle detectors. We detected 130 nm sized magnetic nanoparticle clusters immobilized on sensor surfaces after flowing through PDMS microfluidic channels molded using a 3D printed mold. Two detection schemes were investigated: (i) indirect detection incorporating ferromagnetic antidot nanostructures within microresonators, and (ii) direct detection of nanoparticles without an antidot lattice. Using scheme (i), magnetic nanoparticles noticeably downshifted the resonance fields of an antidot nanostructure by up to 207 G. In a similar antidot device in which nanoparticles were introduced via droplets rather than a microfluidic channel, the largest shift was only 44 G with a sensitivity of 7.57 G/ng. This indicated that introduction of the nanoparticles via microfluidics results in stronger responses from the ferromagnetic resonances. The results for both devices demonstrated that ferromagnetic antidot nanostructures incorporated within planar microresonators can detect nanoparticles captured from dispersions. Using detection scheme (ii), without the antidot array, we observed a strong resonance within the nanoparticles. The resonance’s strength suggests that direct detection is more sensitive to magnetic nanoparticles than indirect detection using a nanostructure, in addition to being much simpler.

## 1. Introduction

Magnetic nanoparticles (MNPs) are employed in a diverse range of novel nanotechnologies, due to their unique magnetic properties and versatile surface chemistry [1,2,3]. MNPs have become particularly useful in medical biosensing, where the nanoparticles bind to analytes such as proteins or viruses and act as markers for immunoassays [2,4,5,6]. For example, magnetic nanoparticle markers have been employed in a number of tests for the SARS-CoV-2 virus [7,8]. In addition, magnetic nanoparticles are used for testing and improving the quality of food, water, and pharmaceuticals [9,10,11,12,13]. There is a growing demand for magnetic nanoparticle biosensors, including portable biosensing devices [7,9].

Biosensing with dispersions of magnetic and nonmagnetic nanoparticles requires sensitive devices capable of detecting single nanoscale objects within complex sample backgrounds [4]. Conventionally, nonmagnetic nanoparticles are detected and counted using luminescence techniques in which the particles are identified by observing their luminescent surface coatings [10,14,15,16]. However, the interference produced by the sample’s background often limits the sensitivity of these devices and must be eliminated using time-based techniques or alterations of the analytes or nanoparticle labels [17,18]. Magnetic nanoparticles can be separated from the complexity of the sample and captured within a sensing area using the magnetophoretic forces generated by localized magnetic field gradients [19,20]. For example, Sun et al. have developed an MNP detector that employs an external permanent magnet to direct magnetic nanoparticles through a microfluidic device and onto a sensing surface [21]. Switching to magnetic particles also enables alternative detection methods based on MNP-induced changes in a static magnetic property such as magnetoresistance (MR) [5,22,23,24,25]. Magnetoresistive detection of MNPs has been demonstrated in a wide variety of devices using techniques such as giant magnetoresistance (GMR), tunneling magnetoresistance (TMR) and anisotropic magnetoresistance (AMR) sensing [5,22,26,27,28]. Of these techniques, GMR has dominated research into MNP sensing. GMR sensors operate by recording changes in the resistance to a current passing through multiple ferromagnetic layers separated by thin nonmagnetic layers. These changes are caused by the magnetic fields generated by MNPs caught on the surface of the multilayered sensor, which perturbs the orientation of the static magnetization of each layer. Nanoparticle detection using MR techniques is generally more accurate than luminescence detection since almost all fluid samples possess a negligible magnetic background; hence, MR sensors typically possess signals with less noise [5,29]. Due to this, MR sensors have achieved very fine accuracies in relatively simple, compact, and cheap packages [30]. However, MR sensors are often heavily dependent upon the ambient temperature and cannot operate within the strong magnetic fields used for magnetic particle separation. An alternative method involving the dynamic properties of the magnetization, such as spin waves and ferromagnetic resonance (FMR), may be capable of accurately detecting MNPs even in these conditions [31,32,33].

Chatterjee et al. proposed an alternative magnetic nanoparticle detection method based upon observation of the ferromagnetic resonances excited within nanoparticle aggregates in a dispersion [34]. Observing changes to the dynamic magnetization of the sensor reduces the 1/*f* noise in the sensing measurements, which increases the potential sensitivity of the device [22]. Metaxas and Sushruth suggested that individual nanoparticles could be detected indirectly, by observing changes in the ferromagnetic resonances (FMRs) of a nanopatterned ferromagnetic thin film rather than resonances in the particles themselves [31]. In these devices, the magnetic fields of the nanoparticles perturb the dynamics of standing spin waves excited within an antidot nanostructure (AN), causing the ferromagnetic resonances to occur at shifted resonance frequencies. Observation of these shifts can be used to imply the presence of magnetic nanoparticles. The magnitude of the shifts in resonance frequency is proportional to the concentration of the nanoparticle dispersion. Using this, one can estimate the number of magnetic nanoparticles present in the dispersion, and the number of analytes bound to them. In addition, more nanoparticles are caught by the sensor due to the magnetophoretic forces generated by the ferromagnetic nanostructure. Sushruth found that this method was more precise, detecting a 0.1 mg/mL dispersion of 150 nm nanoparticle clusters using an array of antidots [31]. Numerical modeling by Manzin et al. indicates that FMR-based detection of MNPs is also possible in rhomboidal arrays of circular antidots [35]. Currently, FMR-based MNP detectors are much larger and more expensive to produce than MR sensors. However, the bulky external equipment required for the sensing measurements is expected to be replaced by microelectronic components and integrated with the microresonator chip, reducing both the price and size. Two steps in this direction will be discussed in this paper. The method developed by Sushruth can be further improved using a planar microresonator. Microwaves traveling through a planar microresonator ring can be used to excite ferromagnetic resonances in nanoscale ferromagnets; hence, on-chip detectors could be designed with significantly smaller sensing areas [36,37]. Integrating these sensors with microfluidics will further reduce the size of the devices and enable integration of the sensors into lab-on-chip devices [38,39,40]. In addition, introduction of the nanoparticles via a microfluidic channel has been shown to increase the amount of nanoparticles caught within the antidot holes, which increases the shifts in resonance field [41]. 

We have fabricated three resonance-based magnetic nanoparticle detectors, each employing a planar microresonator. The first device was used to attempt direct detection of magnetic nanoparticles using only a microresonator. A microfluidic channel was fabricated using a 3D printed mold and pressed against this microresonator to allow a dispersion of nanoparticles to flow over the sensing nanostructure within the microresonator’s inductive ring that defines the most sensitive detection area. A strong absorption peak associated with resonating MNPs was observed for this sensor, suggesting that direct detection of nanoparticles using microresonators provides a sensitive detection scheme. 

Two more devices were fabricated using the same processes, now including a Permalloy AN within the microresonators. We demonstrated that the AN captures MNPs flowing through the microfluidic channel and observed a strong shift in the ferromagnetic resonances of the antidot nanostructure in the second sensor, indicating a successful detection of magnetic nanoparticles. This was compared to the third microresonator device, in which the nanoparticles are introduced in droplets placed onto the surface of the microresonator film, rather than through a microfluidic channel. After depositing multiple droplets, estimates for the sensitivity of this sensor to the mass of MNPs can be made. Introducing the nanoparticles using microfluidics produced far stronger shifts in the resonances than introducing the particles via droplets.

## 2. Materials and Methods

### 2.1. Fabricating Microresonators

**Figure 1:**
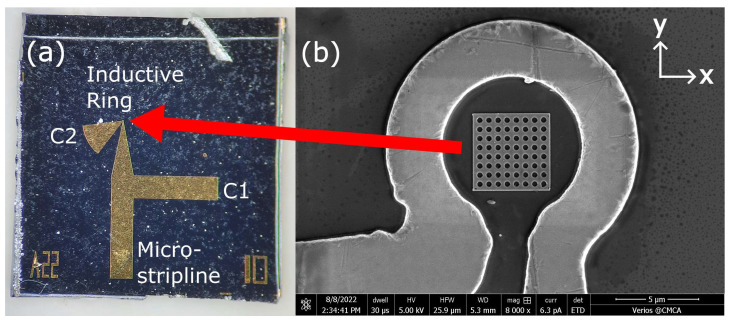
(**a**) An optical microscopy image of a microresonator chip, depicting the microstripline, capacitive stubs C1 and C2, and the inductive ring. The inductive ring for this resonator contains an array of antidots etched into a sheet of Permalloy, shown in further detail in the inset. (**b**) Scanning electron microscopy (SEM) image of the antidot device’s inductive ring surrounding a 50 nm-thick square-shaped sheet of Permalloy. An 8 × 8 array of 400 nm-diameter circular antidots was defined into the 5 × 5 × 0.05 μm^3^ Permalloy sheet using electron beam lithography. The centers of the antidots are separated by 600 nm. The first microresonator device has an identical layout, but no Permalloy antidot nanostructure (AN) inside the ring.

Three copper microresonators were deposited onto highly resistive silicon wafers using deep ultraviolet lithography. One of these microresonators is shown in Figure 1a below. These planar resonators consist of a 20 μm-diameter inductive ring focusing the microwave magnetic field onto the sample, a microstrip line section for connection to a microwave source, and two capacitive stubs that determine the resonant frequency and the impedance matching of the microresonator to the feedline. The microresonator’s ring defines the sensing area, in which the MNPs will be detected. Note that, unlike most MR-based sensors, this sensing area does not need to be coated to immobilize the MNPs for sensing measurements. To connect the sensors to an external microwave circuit, the microresonator chips were bonded to coplanar striplines using silver paste and microscopic gold wiring. Inside the second and third resonator rings, a 50 nm-thick 5 × 5 μm^2^ layer of Permalloy (Ni_80_Fe_20_) was deposited and an 8 × 8 array of circular antidots with 400 nm-diameter was defined using electron beam lithography and lift-off, as shown in Figure 1b. Permalloy was chosen due to possessing FMRs with narrower linewidths and high resistance to corrosion, which allows for prolonged contact with aqueous samples before degradation of the sensor.

**Figure 2:**
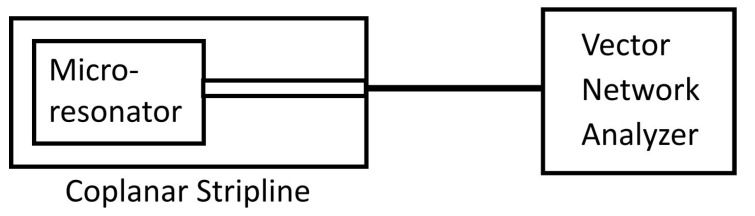
The setup used to identify the resonance frequency of each microresonator and match the microresonators’ impedances to 50 Ω. Each microresonator is bonded to a coplanar stripline that is connected to one port of a vector network analyzer (VNA). A 50 Ω reference resistor is connected to the other port.

Each device was briefly connected to a vector network analyzer (VNA) to observe the resonance peaks for each microresonator, as shown in Figure 2. A small piece of silicon wafer was glued to one of the capacitive stubs of each microresonator to fine tune the microresonators’ impedances to 50 Ω. From the resonance peaks in the spectra, we obtained the resonance frequency of each microresonator: 13.46 GHz for the first resonator, 13.559 GHz for the second, and 13.1 GHz for the third. Using the FWHM linewidths of the microresonator resonance peaks, we also estimated the quality factors of the microresonators as 70.4, 76.6, and 33.6, respectively. These Q-factors are comparable to or slightly higher than the Q-factors for similar microresonators, which lie in the range of 20–30 [42,43].

### 2.2. Fabricating Microfluidic Films

**Figure 3:**
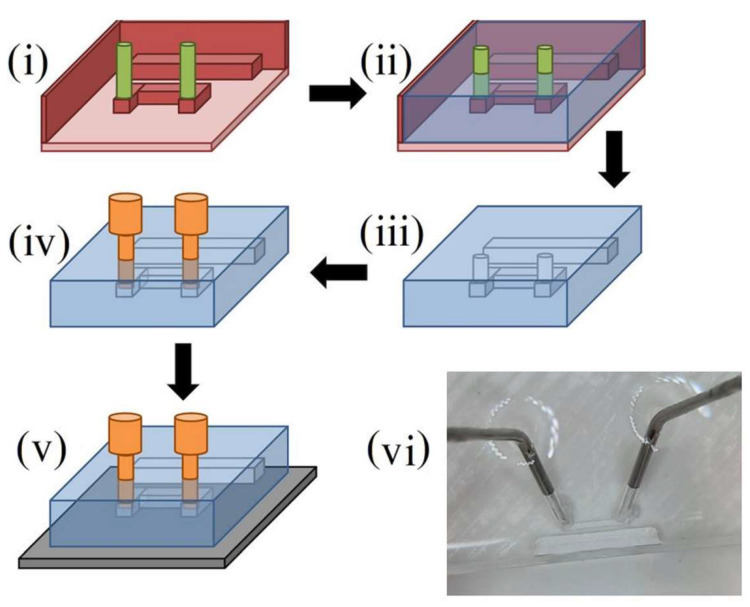
The molding process used to fabricate two open microfluidic channels from PDMS elastomer. (**i**) The U-shaped microchannel mold (red) is 3D printed with PLA thermoplastic. The channel is surrounded by a rectangular wall that contains the PDMS. The stub connected to the wall creates a space for the silicon piece used for impedance matching the microresonator. (**ii**) PDMS elastomer (blue) is poured into the mold and left for 24 h to set. (**iii**) The elastomer is removed from the mold. (**iv**) Luer stubs (orange) are connected to the inlet and outlet arms of the microchannel. This diagram is not to scale. (**v**) The microfluidic film is pressed against the surface of the microresonator film, closing the microfluidic channel. (**vi**) A photograph of the fabricated microchannel.

**Figure 4:**
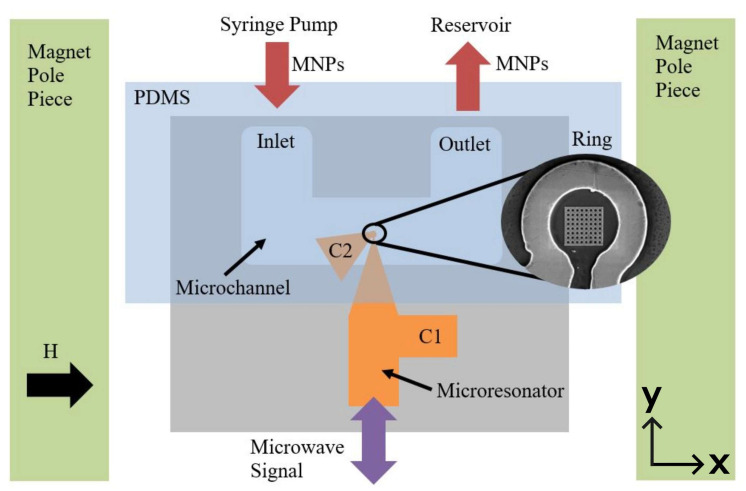
Diagram of the MNP detector showing the microresonator (orange) with two capacitive stubs C1 and C2. The inset shows the microresonator ring containing the antidot array nanostructure. The microfluidic channel (blue) is centered over the ring. The device is placed between two magnetic poles (green) providing an external magnetic dc field *H* along the plane of the detector and parallel to the microchannel. This diagram is not to scale.

Two 4 mm-long microfluidic channels with 400 μm-wide and 400 μm-deep square cross-sections were fabricated from poly (dimethyl siloxane) (PDMS) elastomer using soft lithographic methods [44,45,46,47,48]. The process employed a 3D printed channel mold as shown in Figure 3 [49]. The mold was printed with polylactic acid (PLA) thermoplastic, at 200 °C, and a Creality Ender 3 V-2 printer (Shenzhen, China) with a 0.12 mm-diameter nozzle. The PDMS elastomer base (Sylgard^®^ 184 silicone elastomer, Sigma-Aldrich Pty Ltd., St. Louis, MO, USA) and curing agent were poured into the mold in a ratio of 10:1 by volume and left to set over a period of 24 h in ambient laboratory conditions. Once set, the PDMS sheet was lifted out of the mold and luer stubs (Instech Laboratories, Inc., Plymouth Meeting, PA, USA) were inserted into the inlet and outlet slots to allow connections with external tubing. The microchannels were carefully centered over the microresonators’ rings using an optical microscope and pressed onto the film’s surface using 3D printed clamps, producing an air-tight seal and closing the channels. Sealing the channels using only pressure allowed the channels to be easily removed afterwards for imaging of the nanoparticles and nanostructure. The channel inlets were connected to a syringe pump (Pump 11 Elite, Harvard Apparatus, Holliston, MA, USA) and the outlets were connected to empty reservoirs. Figure 4 shows a diagram of the completed device. Distilled water was then pumped through the devices to ensure the channels were unobstructed and watertight.

### 2.3. Obtaining FMR Spectra before Introduction of MNPs

**Figure 5:**
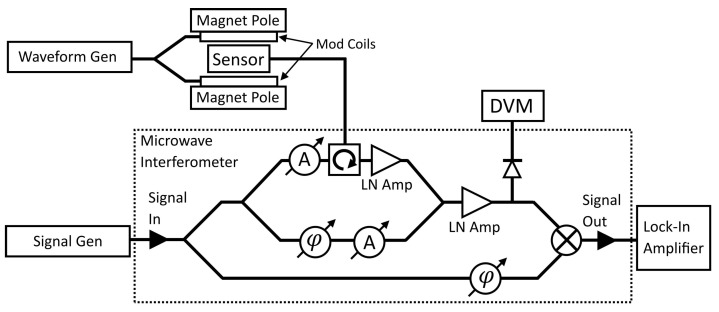
The setup used to observe FMR in the sensors via broadband FMR spectroscopy [50,51]. Each sensor is placed between the poles of an electromagnet producing a static magnetic field in the plane of the sensor. A waveform generator sends an ac current through modulation coils attached to each pole, adding an ac component to the static magnetic field. The sensor is bonded to a coplanar stripline, which is connected to a microwave interferometer (or receiver) via a circulator. A signal generator feeds a microwave signal into the interferometer at the microresonator’s resonance frequency. Attenuators (A), phase shifters (φ), and low noise amplifiers are used to amplify the microwave signal returning from the sensor while maintaining a suitable signal-to-noise ratio [52]. Tuning of the interferometer is monitored using a digital voltmeter (DVM). A homodyne mixer is used to rectify the microwave signal, which is fed into a lock-in amplifier. The amplifier measures the amplitude of the signal and records these data to a PC for later analysis.

Ferromagnetic resonances that occur within the Permalloy nanostructure and MNPs were probed using broadband FMR spectroscopy [50,51]. A diagram showing the arrangement of the sensor and measurement apparatus can be found in Figure 5. The completed microresonator devices were inserted between two magnetic pole pieces of an electromagnet producing a magnetic field parallel with the microfluidic channel, along the x-axis. The magnetic field was monitored using a magnetometer (Magnet-Physik FH 54, Magnet-Physics Inc., Indianapolis, IN, USA) positioned directly above the resonator ring. Microwaves set to each microresonators’ resonance frequency, with an input power of 1 mW (0 dBm), were sent through the devices and via a homodyne mixer into a dual-phase lock-in amplifier (Stanford Research Systems Model SR850 DSP Lock-In Amplifier, Sunnyvale, CA, USA). The two phase-sensitive detectors of the amplifier were used to measure the amplitude of the rectified microwave signal. A microwave interferometer was connected to the microresonator to improve the signal-to-noise ratio [52]. The external magnetic field was swept from remanence to 6 kG to obtain the field-resolved ferromagnetic resonance spectrum of each device. To further improve the setup sensitivity a 10 V (peak-to-peak) ac magnetic field of frequency 1 kHz was added in parallel to the static applied field. The lock-in amplifier was locked to the frequency of this modulation field. The obtained FMR spectra were fit to a series of complex-valued first-derivatives of Lorentzian functions (which represent absorption and dispersion of the microwave signal) and were later used as references to calculate the shifts in the excitation fields of the resonances due to the presence of magnetic nanoparticles [51].

### 2.4. Sensing MNPs Passing through Microfluidic Channels

Having recorded the FMR of the clean microresonator devices, they were now ready for MNP sensing. An aqueous dispersion of MNPs was pumped through each microchannel while changes to the microwave signal were observed using the setup shown in Figure 4 and Figure 5. However, during these measurements, the external magnetic field was kept constant at 4.2 kG, close to the 4 kG at which the MNPs were found to resonate in micromagnetic simulations using MuMax^3^ [53,54]. Measuring the signal 200 G off the resonance peak, where the peak gradient is steeper, resulted in a sharper change in the microwave signal when the MNPs begin resonating. A simplification of this method will most likely be implemented in fully realized sensors. In practice, once a suitable FMR mode has been located in the spectra (such as an extended mode), the magnetic field does not need to be swept. The corresponding static resonance field can be generated by a small permanent magnet or electromagnet. The amplitude of the microwave signal reflected from the microresonator can then be monitored for changes, which indicate MNPs are shifting the resonance field, using a diode and DVM—no lock-in amplifiers or interferometers are required.

Before infusing the nanoparticle dispersion into a microfluidic channel, the syringe pump forced distilled water through the channel at a constant flow rate of 0.3 mL/min for 10 min. Because the MNP dispersion and distilled water contain trace impurities, their dielectric constants will be non-negligible. When the microresonator is wet, this residual conductivity will leech power from the microwave signal. The drop in the signal power could be mistakenly attributed to the magnetic nanoparticles. By introducing distilled water, which will also possess some residual conductivity, into the channels first, the signal amplitudes can reach new stable values before the nanoparticle dispersions begin flowing. Soon after the distilled water came into contact with the microresonator, the microwave receiver was tuned to maximize the signal-to-noise ratio.

Once the infusion of distilled water was complete, the syringe pump forced a 0.1 mg/mL (10^−4^ ppm by mass) dispersion of 130 nm-diameter iron-oxide MNP clusters (Nanomag^®^-D, Micromod Partikeltechnologie GmbH, Rostock, Germany) through the microfluidic channel at a constant flow rate of 0.1 mL/min for 30 min. The nanoparticle dispersion was purchased with a concentration of 25 g/mL that was diluted to 0.1 mg/mL using distilled water. As the particles flowed through the channel, many were caught by the microresonator and exposed to the microwave signal, which excites FMR in the MNPs. The signal for signs of absorption caused by resonating MNPs.

### 2.5. Obtaining FMR Spectra after Introduction of MNPs

Once an infusion of MNPs was complete, 3 mL of distilled water and then 3 mL of air were pumped though the device at 10 mL/min and 3 mL/min, respectively, to clean and dry the microchannel. The FMR spectra of the devices were recorded again using the method outlined in Section 2.3. The external magnetic field was swept and the signals from the lock-in amplifier were recorded. The new responses were compared with the clean FMR responses to determine the magnitude of any shifts in the excitation fields of the FMRs. The microchannel films were then removed and the surfaces of the microresonators were then imaged using SEM to observe the location and quantity of nanoparticles caught on the surface.

### 2.6. Sensing MNPs in Microresonator Device without Microfluidics

The third microresonator device contained an AN but was not pressed against a microfluidic film. For this film, the MNPs were introduced to the sensor via droplets deposited onto the surface of the microresonator film [55]. The dispersion of 130 nm MNP clusters was diluted from the original concentration of 25 g/mL to 2 μg/mL using distilled water. Following the method outlined in Section 2.3, the FMR spectrum of the microresonator was obtained before and after dropping a 0.5 μL droplet of the 2 μg/mL dispersion onto the microresonator’s inductive ring via a pipette, under an optical microscope. The droplet was left to evaporate in ambient laboratory conditions. The two spectra were compared to observe any changes caused by the addition of magnetic nanoparticles. This process was repeated five more times to observe the FMR as the effective concentration of the MNP dispersion droplet increased from 0 to 12 μg/mL.

## 3. Results and Discussion

### 3.1. Detecting MNPs with a Bare Microresonator

**Figure 6:**
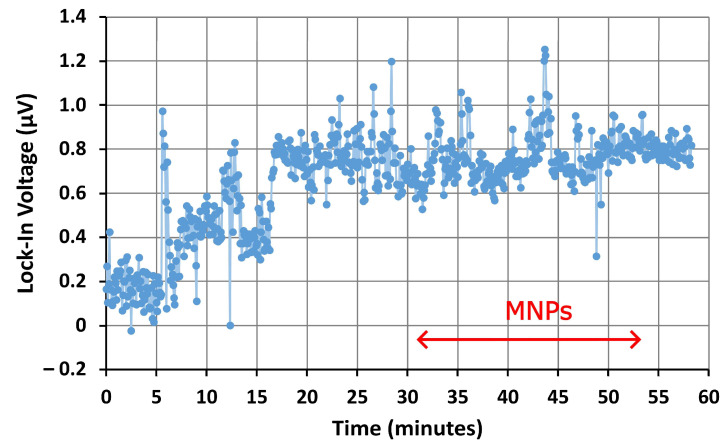
Voltage from the first device with a bare microresonator during the flow of MNPs through the tubing and microfluidic channel. The double-headed arrow indicates the approximate period of time in which the MNP suspension was flowing through the microfluidic channel. During this time, any changes in the signal may indicate the presence of MNPs.

The signal from the lock-in amplifier during the pumping of magnetic nanoparticles through the microfluidic channel of the first device, without the AN, is displayed in Figure 6. There was an unstable period over the first 20 min during which distilled water was pumped into the channel. Then, the signal was stabilized. Fluctuations in the signal are likely due to residual conductivity in the distilled water, which removes power from the microwave signal traveling through the microresonator’s exposed inductive ring. As the pressure of the water varies slightly during infusion, this caused the amplitude of the signal to fluctuate slightly. The sensitivity of the devices to water could be used for other applications, such as monitoring for moisture in electronics and wearable devices [56]. However, for nanoparticle detection, these fluctuations are undesirable. Improving the uniformity of the microfluidic flow by redesigning the geometry of the microchannel may reduce these fluctuations.

**Figure 7:**
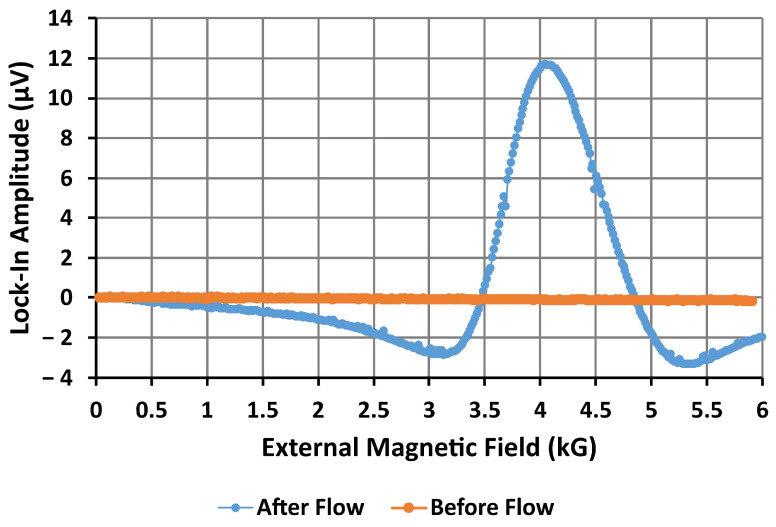
Microwave signal amplitude from the first sensor (microresonator without antidot array) before and after MNPs flowed through the microfluidic channel. The external magnetic field was swept from remanence to 6 kG to obtain the ferromagnetic resonance spectrum for this bare device. The resonance of the MNPs can be clearly observed in the signal obtained after pumping the MNPs through the channel.

Once the signal became stable, the infusion of magnetic nanoparticles was started, with the particles reaching the microfluidic channel after approximately 31 min. No appreciable changes in the signal, which indicates the presence of magnetic nanoparticles, were observed. This implies that real-time sensing of nanoparticles flowing through a microfluidic channel is not possible using this device in its current design. However, a strong change was observed in the FMR spectra (shown in Figure 7) obtained after the device had dried, which suggests that the influence of the MNPs is reduced when they are floating in the dispersion. Because the microchannel, with a width and height of 400 μm, is an order of magnitude larger than the inductive ring, with a diameter of 20 μm, most of the particles moving through the channel are far from the influence of the microwave traveling through the resonator ring. The microresonator excites resonances only in the relative few nanoparticles traveling close to the ring. The MNP resonance therefore has a reduced amplitude and is hidden by the noise generated in the microresonator and external circuitry. This indicates that, if the microresonator ring is exposed to an aqueous sample, the depth of the microchannel must be reduced to make real-time detection of magnetic nanoparticles possible. When the channel dries, the nanoparticles present within the microchannel are deposited onto the surfaces of the channel and the microresonator. An image obtained using SEM showing MNPs deposited onto the surface of the bare microresonator’s ring can be found in Appendix A. Since more particles are now caught within the resonator ring, the MNP resonance should be stronger and easier to observe. Another potential solution is to increase the diameter of the microresonator’s ring [42]. By increasing the diameter of the microresonator’s ring, a greater number of magnetic nanoparticles will pass through the microwave magnetic field generated by the ring, which will resonate and contribute to the sensing signal. This should increase the signal-to-noise ratio, allowing for changes to the signal to be observed during the pumping of MNPs through the microchannel. However, focusing the magnetic field over a larger area will also decrease the magnetic flux density within the ring, resulting in weaker resonances in the nanoparticles. If the sample contains a high concentration of MNPs, the decrease in resonance amplitude will be compensated by the increase in resonating nanoparticles. However, for samples with dilute concentrations of MNPs, the increased number of resonating nanoparticles may be insufficient to compensate for the reduction in the resonance amplitudes. In this case, it may be worthwhile to focus on reducing the dimensions of the microfluidic channel rather than the microresonator ring.

The FMR spectra obtained from the bare resonator before and after pumping MNPs across the microchannel have been plotted in Figure 7 below. Nothing can be observed in the spectrum before pumping, since there is no ferromagnetic material near the resonator’s inductive ring. After pumping the MNP dispersion, the resonance of the magnetic nanoparticles is clearly observable at the predicted external magnetic field of 4 kG, indicating the presence of MNPs. The absorption peak has a strong amplitude of 12 μV, making it easily identifiable among the noise. This indicates that a bare microresonator is capable of detecting a dried 10^−4^ g/mL dispersion of magnetic nanoparticles. However, the MNPs could not be sensed while the nanoparticles were in suspension and flowing through the channel. Detection of weaker dispersions or single nanoparticles as they pass through the device may be possible with improvements to the design of the sensor. Further testing over a range of dispersion densities will be required to obtain an estimate of the sensitivity of the device and the device’s limit of detection.

### 3.2. Detecting MNPs with an Antidot Microresonator

Before probing the FMR of the microresonator devices with antidot nanostructures, the FMR spectrum of an AN was numerically modeled using Mumax^3^. In the model, the AN was comprised of a square 8 × 8 array of 400 nm circular antidots separated by 600 nm between the centers of each antidot, hollowed into a 5 × 5 × 0.05 μm^3^ square sheet. The sheet was given an exchange stiffness of $13\times 10^{-12}\;J/m$, a saturation magnetization of 800 kA/m, and a gyromagnetic ratio of $1.85\times 10^{11}\;\mathrm{Hz}/T$. A static magnetic field of 4 kG was applied in the plane of the AN, along the x-axis. An AC magnetic field, which drives the FMR, was directed along the z-axis, the direction of the magnetic field generated by the microwave current passing through the microresonator. 

**Figure 8:**
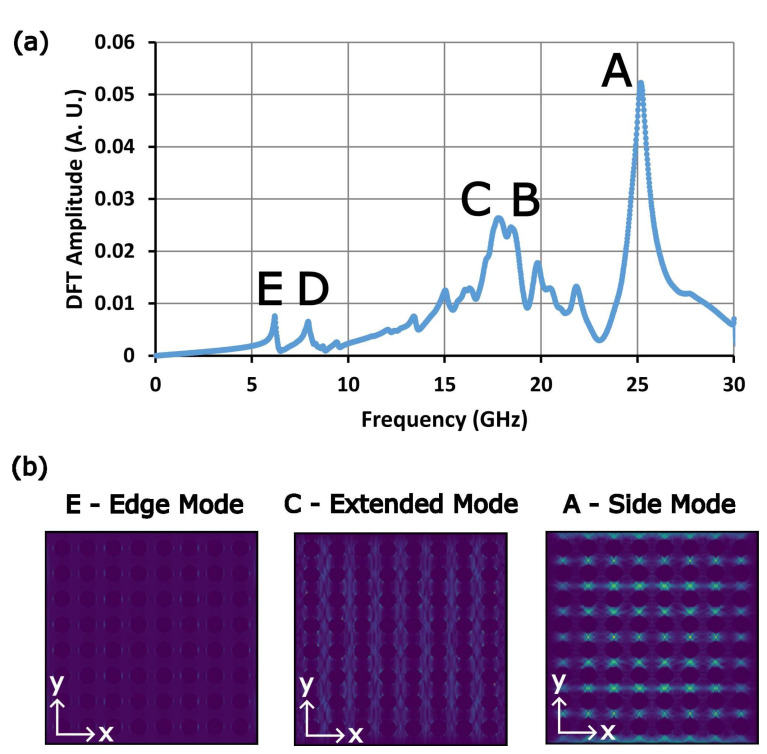
(**a**) Ferromagnetic resonance spectrum for an 8 × 8 array of 400 nm-diameter circular antidots numerically modeled using MuMax^3^. The external static magnetic field was kept at 4 kG and directed along the x-axis. The frequency of the microwave excitation was swept up to 30 GHz to obtain a frequency-resolved ferromagnetic resonance spectrum for the ANs employed in these sensors. A single-side mode labeled A is present in the spectrum, along with two strong extended modes labeled B and C. The A, B, and C modes are analogous to the modes observed in the second device’s FMR spectrum shown in Figure 10. For the third device, only the A mode and one of the extended modes can be observed in the spectrum shown in Figure 11. The two edge modes labeled D and E on the far left were not visible in either experimental spectrum. (**b**) The spatial distributions of the ferromagnetic resonance modes that occur at each of the peaks labeled A, C, and E in the ferromagnetic spectrum of the 8 × 8 circular antidot array. Each resonance mode is localized within a different region of the antidot nanostructure.

The simulated spectrum shown in Figure 8a indicates the presence of five prominent resonance modes in the AN, labeled A to E. As shown in Figure 8b, these resonances occur within different regions of the ferromagnetic nanostructure [31,57,58]. At the highest resonance frequency is the A mode, which is localized within the horizontal strips between the antidots, parallel to the external magnetic field. This mode is usually called a “side” mode. The next prominent modes, B and C, are localized within the vertical strips between the antidots, perpendicular to the external magnetic field. These modes have been referred to as “extended” modes. Finally, the D and E modes at the lowest resonance frequencies are located along the edge of each antidot. These modes are referred to as “edge” modes. Because their resonance fields are so high, and they are localized within such small areas, the edge modes are often difficult to observe experimentally.

**Figure 9:**
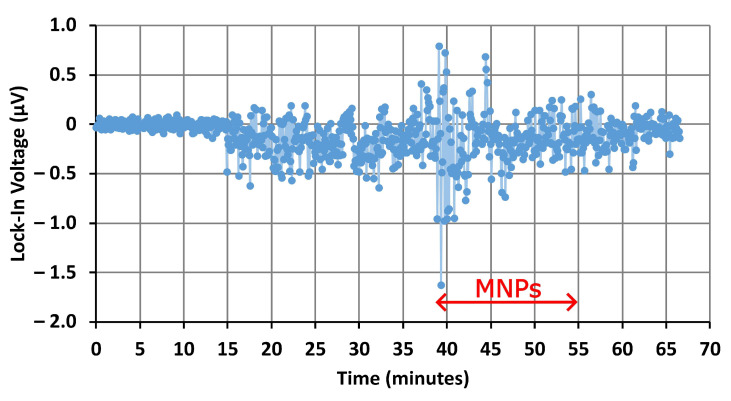
Voltage from the second device (microresonator with AN and microfluidics) during the flow of MNPs through the tubing and microfluidic channel. The double-headed arrow indicates the approximate period of time in which the MNP suspension was flowing through the microfluidic channel. During this time, any changes in the signal may indicate the presence of MNPs.

We will now discuss the experimental results obtained for the second device, in which the nanoparticles are introduced to an AN via a microfluidic channel. The microwave signal measured by the lock-in amplifier during the pumping of magnetic nanoparticles through the microfluidic channel of the antidot device is displayed in Figure 9. Only air was present within the channel for the first 15 min as the distilled water was pumped through the tubing and into the microchannel. When the distilled water reaches the resonator ring, the residual conductance of the water again absorbs power from the microwave signal, causing the noise to suddenly increase. Compared to the signal from the first device without an AN, the microwave signal of the second device with an AN appears to be relatively stable once the resonator ring is exposed to water. As with the first device, no changes were observed as the MNPs were forced over the resonator’s ring. This is further evidence that the current design, with the resonator’s ring exposed to water, cannot be used to detect magnetic nanoparticles in real-time.

An image of the distribution of MNPs that formed on the surface of the AN in the second device, once all of the water had evaporated, can be found in Appendix B. Although the amplitudes of the resonances are lower than expected, reaching a maximum amplitude of only 0.4 μV, three strong absorption peaks are present in the FMR spectra obtained before and after introducing magnetic nanoparticles to the sensor. The structures of the spectra shown in Figure 9 and the simulated spectra shown in Figure 8a share several similarities. The first peak in the physical spectra occurs at 2.96 kG and can be identified as the side mode A. The other two modes occur relatively close together, at 3.71 kG and 3.89 kG. Based upon their proximity, amplitude, and position relative to the side mode, these two peaks can be identified as the extended mode resonances B and C, respectively. No edge modes were observed in any of the spectra. Compared to the modeled resonances, these modes occur at significantly higher magnetic fields, 2.96 kG and 3.89 kG for the A and C modes, respectively. Coincidentally, mode C now occurs close to the expected position of the MNP resonance, at 4 kG. We suspect that the perpendicular magnetic anisotropy in the bulk of the Permalloy is higher, which has resulted in the higher resonance fields. From a practical view, this is not an issue, as we only need to observe shifts in these resonance fields to sense the presence of MNPs. Unlike the bare resonator, the antidot resonator showed no discernable MNP resonance around 4 kG. However, the amplitude of mode C has increased noticeably, while the amplitude of nearby mode B has not. Since the MNP and C modes would only be separated by approximately 100 G, resonance mode C appears to have hidden the MNP resonance.

The low resonance amplitudes are likely due to poor impedance matching between the microresonator and external microwave circuitry, producing poor microwave coupling to the microchannel. The impedance of the water, microchannel, and external circuitry, which were not considered at the time, may also contribute to the total impedance of the device. Poor coupling reduces the power delivered to the nanoparticles, reducing the amplitude of the resonance absorption peaks. Optimization of the impedance matching of the microwave power to the microresonator would enhance the amplitudes of the resonances. A stronger signal-to-noise ratio makes the resonances easier to locate, which may improve the accuracy of future devices. However, this is only a technical issue since the positions of the resonance peaks can be located with accuracy sufficient for sensing the presence of magnetic nanoparticles.

**Figure 10:**
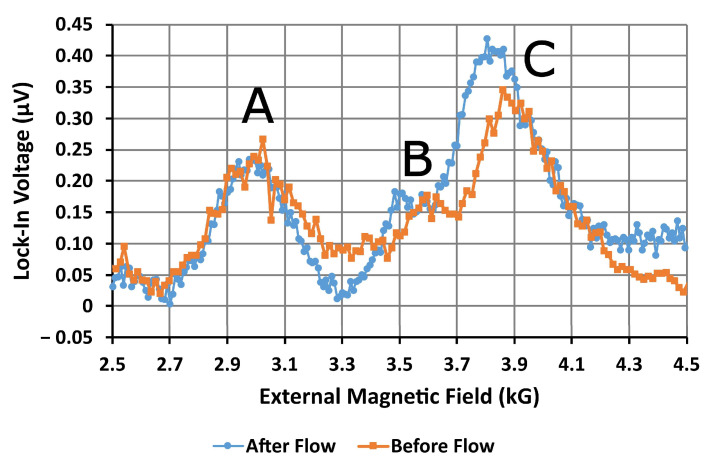
Microwave signal amplitude from the second device (microresonator with AN and microfluidics) before and after MNPs flowed through the microfluidic channel. The external dc magnetic field was swept from remanence to 6 kG to obtain the field-resolved ferromagnetic resonance spectrum for the device. The spectrum contains three ferromagnetic resonance modes labeled A, B, and C. After flowing MNPs, the B and C resonances occur at lower magnetic fields. Note that, since the simulated spectrum was frequency-resolved, the order of the modes appears reversed in this field-resolved spectrum.

When the two measured FMR traces are plotted together, as shown in Figure 10 below, the B and C extended mode resonances are seen to occur at noticeably lower resonance fields after introducing the nanoparticles. The positions of all three resonance modes were extracted from fits of both traces to the derivatives of complex-valued Lorentzian functions. The shifts in each of the three modes have been collected into Table 1. Mode B shifted down-field by 207 G and mode C shifted down-field by 128 G. These shifts are considerably larger than the largest shifts observed by Metaxas and Sushruth, which were approximately 120 G [31]. Shifts of this magnitude are easily sufficient for sensing magnetic nanoparticles. Mode A also shifted down-field by 12 G, but this difference is far too small for practical sensing. Observation of shifts in the positions of the resonances confirms that this method can be used to detect magnetic nanoparticles.

### 3.3. Detecting MNPs with an Antidot Microresonator without Microfluidics

**Figure 11:**
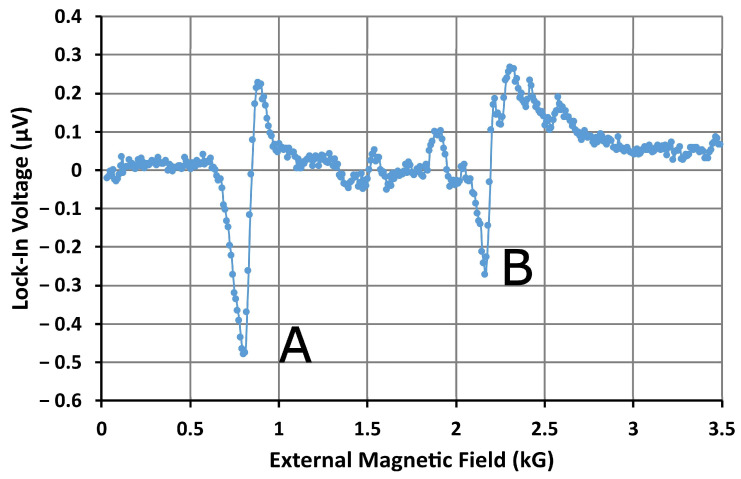
Microwave signal amplitude from the third device (microresonator with an AN, no microfluidics) before MNPs were introduced to the surface of the microresonator. The external dc magnetic field was swept from remanence to 6 kG to obtain the field-resolved ferromagnetic resonance spectrum for the device. The spectrum contains two prominent ferromagnetic resonance modes labeled A and B. Note that, since the simulated spectrum was frequency-resolved, the order of the modes appears reversed in this field-resolved spectrum.

Sensing was also demonstrated in a third device with identical microresonator and AN layouts, but without microfluidics. The FMR spectrum for this device’s AN, taken before introducing MNPs onto the sensor’s surface, is shown in Figure 11. In this spectrum, we observe two clear absorption peaks. The first peak occurs at 815 G and can be identified as the side mode A. The second peak occurs at 2.19 kG and is identified as an extended mode, which will be labeled B. The amplitudes of these resonances are considerably larger than those for the first AN. The side mode A has an amplitude, from peak to peak, of approximately 0.7 μV—almost twice the amplitude of extended mode C in the second sensor. In addition, the resonance fields in the third sensor are considerably lower, with the extended and side modes occurring at 2186 G and 815 G, respectively, compared to 3.89 kG and 2.96 kG for the second sensor. These resonance fields are closer to the values predicted in the numerical model and previous measurements of the FMR for antidot nanostructures in similar static magnetic field configurations [31]. However, an MNP resonance was not observed in the spectrum from this device. This is most likely due to the concentration of MNPs in the droplets (2 μg/mL) being much smaller than the concentration of the dispersion flowing though the microfluidic channel (100 μg/mL). Since fewer MNPs are present in the sensing area, the amplitude of the FMR peak produced by the MNPs is much weaker.

Magnetic nanoparticles in 0.5 μL droplets of a 2 μg/mL dispersion placed onto the surface of the microresonator film, directly over the microresonator ring. The droplets were then left to evaporate in ambient laboratory conditions, leaving only MNPs caught on the surface. Once the droplet had completely dried, the FMR was observed using the same method as the previous two microresonator devices. Since there were relatively few MNPs in the droplet, the shift in the resonance fields of both the resonance modes was almost imperceptible, at only 1–2 G. However, as more droplets were evaporated on the surface, the resonance field shifts increase in magnitude.

**Figure 12:**
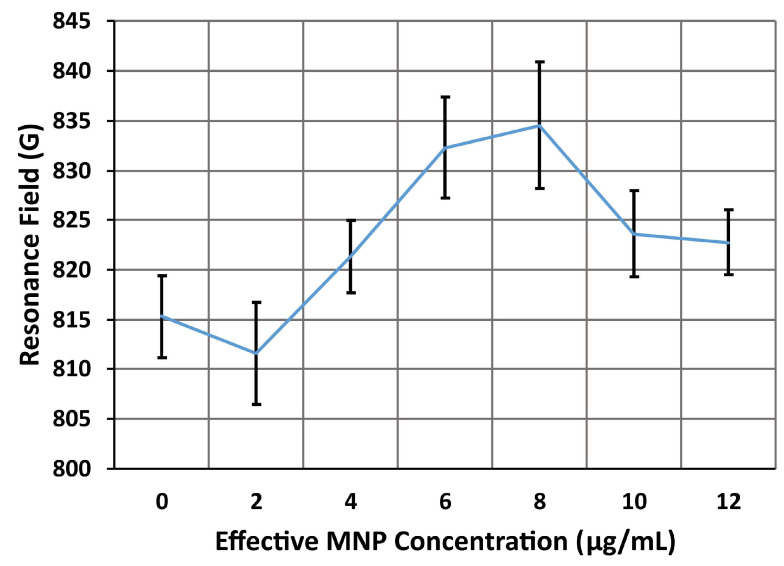
The resonance field of the side mode, A, of the third device (microresonator with AN, no microfluidics) as the effective concentration of the MNP dispersion droplet increases from 0 to 12 μg/mL. Initially, the resonance field increases with the droplet concentration, reaching a maximum shift of 20 G at 8 μg/mL. The resonance field then begins to decrease slightly, the final shifts being only approximately 8 G. Each of the points in this plot was averaged over six measurements of the FMR spectrum, with the error bars indicating the standard deviation of these six measurements.

For the side mode, A, shown in Figure 12, the resonance field gradually increased from 815 G and peaked at almost 835 G at an effective droplet concentration of 8 μg/mL. The greatest shift observed in the side mode is therefore approximately 20 G—comparable to the 12 G shift observed in the resonance field for the side mode in the second device, with MNPs introduced via a microfluidic channel. However, the side mode in the third device shifts upwards in field, which is consistent with the shifts observed in numerical models and prior experiments [31]. Fitting the data up to an effective concentration of 8 μg/mL with a linear trend, we find that the trend is almost linear, with a coefficient of determination of *R*^2^ = 0.85. The slope of this trend, which is indicative of the sensitivity of the device, is 5.91 GmL/μg. Taking into account the volume of each droplet, roughly 500 nL, we can convert the effective concentration of the droplet to the total mass of MNPs introduced to the sensor. With this, we obtain an estimate for the sensitivity of the side mode’s resonance field to the mass of MNPs applied to the sensor: 5.91 G/ng. 

The resonance field then begins to decrease slightly, reaching a final value of approximately 823 G at an effective droplet concentration of 12 μg/mL. Fitting the entire range of data, up to 12 μg/mL, with a linear trend, the coefficient of determination is now *R*^2^ = 0.31, indicating the trend is much less linear. The sensitivity of side mode A is now 2.13 G/ng, a reduction of 64% from the previous estimate. This nonlinear behavior has been seen in many of the past experiments involving sensing with antidot nanostructures [31]. This effect is likely due to the AN being almost completely covered by MNPs, which spreads the MNP-generated magnetic fields across the entire nanostructure. The magnetic field near the bulk of the MNPs is reduced, causing the shifts in resonance field to plateau [31]. SEM Imaging of the AN, shown in Appendix C, confirmed that the surface had been almost completely covered by MNPs at the effective droplet concentration of 8 μg/mL. 

**Figure 13:**
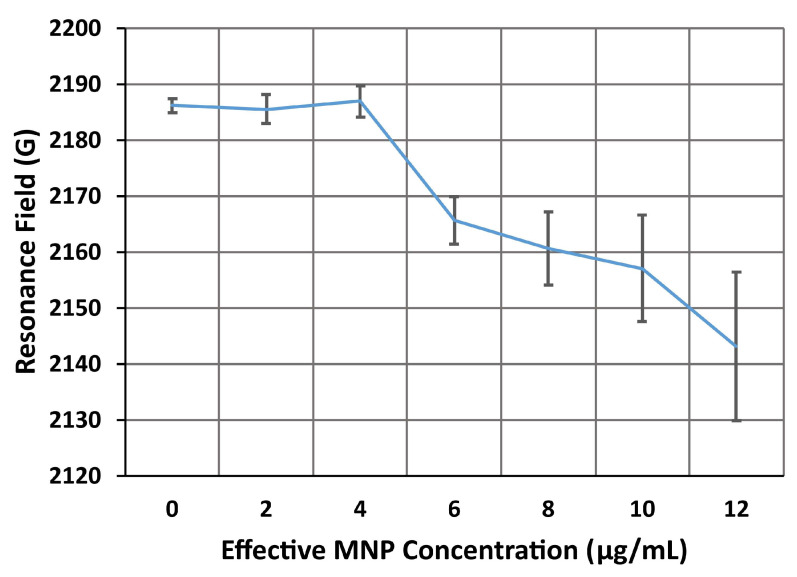
The resonance field of the extended mode, B, of the third device (microresonator with AN, no microfluidics) as the effective concentration of the MNP dispersion droplet increases from 0 to 12 μg/mL. The resonance field steadily decreases with the droplet concentration, reaching a maximum downwards shift of 44 G at 12 μg/mL. Each of the points in this plot was averaged over 6 measurements of the FMR spectrum, with the error bars indicating the standard deviation of these measurements.

As seen in Figure 13, the resonance field for extended mode B in the third device decreased relatively steadily, reaching 2141 G at an effective droplet concentration of 12 μg/mL. From the initial resonance position of 2183 G, the resonance ultimately shifted downwards by 44 G. This shift is significantly smaller than the 128 G and 207 G shifts observed in the extended modes of the second device. This suggests that introducing the MNPs via a microfluidic channel produces larger shifts in the extended mode of the AN. This is probably due to the flow removing most of the particles caught outside of the antidot holes. An external magnetic field was also applied while the MNPs were pumped through the channel. This attracts more nanoparticles into the antidot holes [41] and has been shown to increase the field shift of an AN’s extended mode [31]. However, the shifts are all toward lower resonance fields, which is consistent with prior modeling and experiments [31]. The trend observed between the resonance field and the effective concentration of the droplet was surprisingly linear. Fitting the data for the extended mode to a linear trend, we obtained a coefficient of determination *R*^2^ = 0.9. From the slope of the trend, −7.57 GmL/μg, we can estimate the sensitivity of the extended mode’s resonance field to the total mass of MNPs introduced to the sensor to be roughly 7.57 G/ng. Extended mode B appears to be significantly more sensitive to MNPs than side mode A, with a maximum estimated sensitivity of approximately 5.91 G/ng. The extended mode appears to be the most suitable resonance mode for quantifying the concentration of the dispersion droplet. From this, one can calculate an estimate of the total number of MNPs present in the sample droplet. Considering that the extended modes in both the second and third device also show the largest responses to MNPs, it seems clear that extended modes are ideal for FMR-based sensing of magnetic nanoparticles with antidot nanostructures. 

## 4. Conclusions

Three magnetic nanoparticle detectors employing microresonators were fabricated: the first with a bare microresonator with microfluidics, the second containing a ferromagnetic AN and microfluidics, and the third with the AN but without microfluidics. Using soft lithography and 3D printed molds, microfluidic channels were created and pressed against the first and second microresonators. This molding process is simple and relatively fast, making it ideal for the fabrication of prototype microfluidics. However, the microchannels could be fabricated from PDMS using hard lithographic methods, as an addition to the fabrication process employed to fabricate the microresonators.

Numerical modeling indicates that the ferromagnetic ANs employed in these sensing devices produce four prominent ferromagnetic resonances. This FMR spectrum is typical for ferromagnetic ANs, with each of the resonance modes localized within different regions of the AN. The extended and side resonance modes were identified in the measured spectra of both devices containing an AN. A 10^−4^ g/mL dispersion of 130 nm-diameter magnetic nanoparticle clusters was pumped through the microchannels and the presence of the nanoparticles was detected by observing changes in the resonance fields of the devices. After the dispersion had dried within the microchannel, the extended modes were found to have shifted toward lower magnetic fields by up to 207 G whereas the side mode shifted downwards by only 12 G. Observing such a strong shift in the extended mode demonstrates that a microresonator containing a ferromagnetic AN can successfully sense the presence of magnetic nanoparticles immobilized upon the AN surface. However, the MNPs could not be detected while the MNP dispersion was flowing through the microfluidic channel. This is likely due to the dimensions of the microfluidic channel being an order of magnitude greater than the dimensions of the microresonator’s inductive ring, which defines the sensing area. 

For the third device without microfluidics, the magnetic nanoparticles were introduced onto the microresonator film’s surface suspended within droplets. After multiple droplets had been evaporated on the surface, the shifts in the extended resonance mode of the third device reached only 44 G for an effective MNP dispersion of 12 μg/mL. The resonance field varied linearly with the effective concentration of MNPs in the droplet, with an *R*^2^ value of 0.9. A linear trend will enable easy determination of the quantity of MNPs present in a sample. From the trend in the resonance field, we estimate the sensitivity of the extended mode to the MNP mass to be 7.57 G/ng. The maximum shift observed in the side mode was only 20 G. The resonance field for this FMR mode was initially quite linear, with an *R*^2^ value of 0.85. However, the trend began to plateau after the droplet reached an effective concentration of 8 μg/mL. The sensitivity of the side mode to MNPs was initially 5.91 G/ng, but it dropped to 2.12 G/ng. The side mode is both less sensitive to MNPs and responds less linearly than the extended mode. These results demonstrate that it is also possible to sense MNPs from droplets evaporated on the surface, but the shifts are considerably smaller. More infusions will need to be performed for both of the microfluidic devices with dispersions of weaker MNP concentrations to produce estimates of the sensitivity and limit of detection of these devices. 

After pumping the 10^−4^ g/mL dispersion of nanoparticle clusters into the first device, a strong MNP resonance was observed at 4 kG. This indicates that a simple microresonator device is sufficient for direct detection of magnetic nanoparticles. The strength of this resonance suggests that this device may be very sensitive to magnetic nanoparticles. It may be possible for the device to detect single MNPs through direct observation of their FMR. Like the second device with both an AN and microfluidics, no changes were observed during the infusion of MNPs through the microfluidic channel. Real-time detection of magnetic nanoparticles may be possible if the microchannel dimensions are reduced or the resonator diameter is enlarged. Reduction in the microfluidic channel will bring the MNPs closer to the inductive ring, exciting FMR in more MNPs. This may produce an absorption signal strong enough for real-time detection of MNPs. Compared with the devices containing ANs, the fabrication of this bare microresonator is simpler and sensing only requires identification of a single resonance. This simplicity makes magnetic nanoparticle detection using only a microresonator particularly promising.

## Figures and Tables

**Table 1:** The magnetic fields at which each of the resonance modes A, B, and C occur in the second device’s antidot nanostructure (AN) before and after pumping magnetic nanoparticles (MNPs) through the microfluidic channel. The resonance fields for the B and C modes have shifted noticeably downwards. Observation of shifts such as these can be used to infer the presence and quantity of MNPs.

Ferromagnetic Resonance Fields before and after MNP Flow			
Resonance Mode	Before Flow (G)	After Flow (G)	Difference (G)
A	2962	2950	−12
B	3713	3506	−207
C	3893	3765	−128

**Figure A1:**
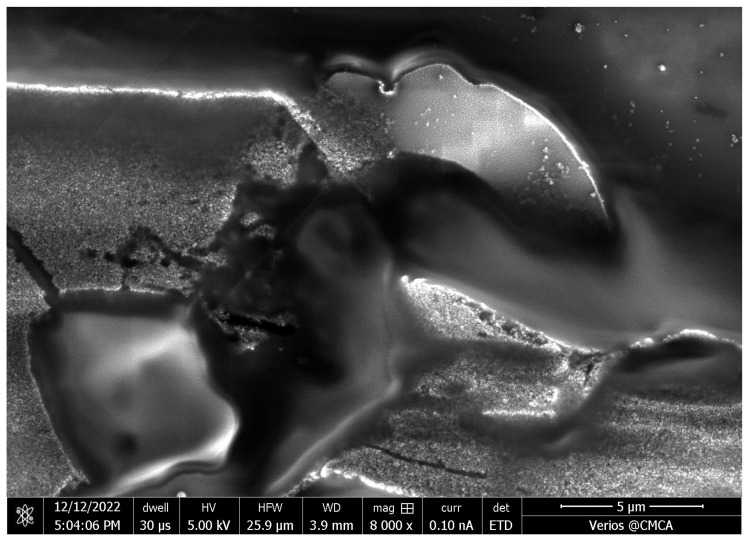
An image obtained using scanning electron microscopy of the first microresonator’s inductive ring after infusion of magnetic nanoparticles. Since the ring was misplaced near the edge of the microfluidic channel, the ring has been partially covered by a large agglomeration of nanoparticles.

**Figure A2:**
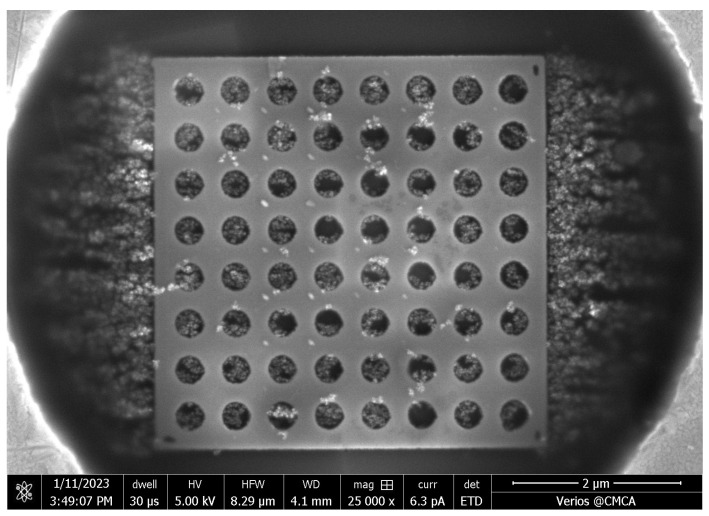
An image obtained using scanning electron microscopy of the antidot nanostructure of the second device after infusion of magnetic nanoparticles. This ring was correctly placed near the center of the microfluidic channel, so it has not been covered in nanoparticles. Most of the particles have been caught within the antidots and along the two edges of the nanostructure perpendicular to the external magnetic field.

**Figure A3:**
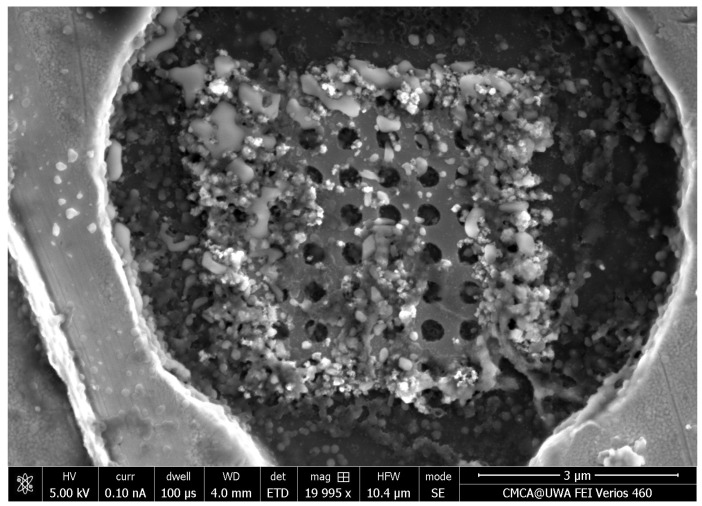
An image obtained using scanning electron microscopy of the antidot nanostructure in the third device after introduction of magnetic nanoparticles onto the surface via droplets. The MNPs have almost completely covered the antidot nanostructure. This image was obtained for an effective droplet concentration of 8 μg/mL.

## Data Availability

The data presented in this study are openly available in the UWA Profiles and Research Repository at https://doi.org/10.26182/jhks-se60, accessed on 27 July 2023.

## Appendix A. Magnetic Nanoparticles Captured by a Bare Microresonator

After the detection of magnetic nanoparticles was confirmed, the microfluidic channel was peeled away from the first microresonator’s surface. The microresonator ring was then imaged using scanning electron microscopy (SEM). Imaging revealed that the microchannel film had been misaligned during its placement onto the microresonator’s surface. This resulted in the resonator ring being located closer to the edge of the microchannel. MNPs were found to have deposited along the edges of the channel, covering most of the microresonator’s ring. An image of this is shown in Figure A1. The large quantity of MNPs within and near the microresonator is the likely cause of the strong absorption peak that was observed in the FMR spectrum. Positioning the resonator ring closer to the edges of the microchannel may therefore be optimal for sensing the presence of magnetic nanoparticles. However, Sushruth found that the shifts observed in the FMR of a ferromagnetic AN saturated and did not increase beyond a dispersion concentration of 6 mg/mL and were difficult to observe below 0.03 mg/mL [31]. Quantification of the concentration of nanoparticles using an AN device may therefore be difficult at higher and lower concentrations of MNPs. Positioning the resonator ring at the edge of the microchannel, covering the resonator ring with nanoparticles, may therefore saturate the microresonator device and impede the device’s ability to quantify the concentration of the dispersion and the number of nanoparticles it contains. This should not be a problem for the first device, without an AN, as the amplitude of the MNP resonance peak in the signal is proportional to the number of particles near the sensing area.

## Appendix B. Magnetic Nanoparticles Captured by an Antidot Microresonator with Microfluidics

After all measurements were complete, the microfluidic channel was peeled away from the second microresonator’s surface. The PDMS microchannel film was correctly placed onto the microresonator chip’s surface, with the resonator ring positioned at the center of the microfluidic channel. The quantity and distribution of magnetic nanoparticles caught within the AN were observed using SEM. An image of the nanostructure is shown in Figure A2. Because the resonator ring is correctly positioned at the center of the microchannel, far fewer nanoparticles have been caught within the antidot resonator ring. The lower density of MNPs may have been a factor in the absence of a MNP resonance in the FMR spectrum obtained for the second microresonator device. Most of the magnetic nanoparticles were captured within the antidots with very few caught on the outer surface of the structure. This distribution of magnetic nanoparticles was predicted in previous works [41,59]. Prior simulations of the magnetophoretic forces suggest that an external magnetic field in this configuration, parallel to the plane of the nanostructure, produces magnetophoretic forces that are attractive only over the antidots and close to the two edges of the structure perpendicular to the applied magnetic field, in agreement to what is seen in Figure A2.

## References

[1] Hasany S.F., Rehman A., Jose R., Ahmed I. (2012). Iron oxide magnetic nanoparticles: A short review. AIP Conf. Proc..

[2] Ali A., Shah T., Ullah R., Zhou P., Guo M., Ovais M., Tan Z., Rui Y. (2021). Review on recent progress in magnetic nanoparticles: Synthesis, characterization, and diverse applications. Front. Chem..

[3] Shipway A.N., Katz E., Willner I. (2000). Nanoparticle arrays on surfaces for electronic, optical, and sensor applications. Chem. Phys. Chem..

[4] Wu K., Su D., Saha R., Wong D., Wang J.-P. (2019). Magnetic particle spectroscopy-based bioassays: Methods, applications, advances, and future opportunities. J. Phys. D Appl. Phys..

[5] Chen Y.-T., Kolhatkar A.G., Zenasni O., Xu S., Randall Lee T. (2017). Biosensing using magnetic nanoparticle detection techniques. Sensors.

[6] Wu C.C., Lin L.Y., Lin L.C., Huang H.C., Yang Y.F., Liu Y.B., Tsai M.C., Gao Y.L., Wang W.C., Hung S.W. (2008). Biofunctionalized magnetic nanoparticles for in vitro labeling and in vivo locating specific biomolecules. Appl. Phys. Lett..

[7] Pietschmann J., Voepel N., Voβ L., Rasche S., Schubert M., Kleines M., Krausse H.-J., Shaw T.M., Spiegel H., Schroeper F. (2021). Development of fast and portable frequency magnetic mixing-based serological SARS-CoV-2-specific antibody detection assay. Front. Microbiol..

[8] Wu K., Saha R., Su D., Krishna V.D., Liu J., Cheeran M.C.-J., Wang J.-P. (2020). Magnetic-nanosensor-based virus and pathogen detection strategies before and during COVID-19. ACS Appl. Nano Mater..

[9] Luo Y., Alocilja E.C. (2017). Portable nuclear magnetic resonance biosensor and assay for a highly sensitive and rapid detection of foodborne bacteria in complex matrices. J. Biol. Eng..

[10] Li C., Hai J., Li S., Wang B., Yang Z. (2018). Luminescent magnetic nanoparticles encapsulated in MOFs for highly selective and sensitive detection of ClO-/SCN- and anti-counterfeiting. Nanoscale.

[11] Fallahianbijan F., Giglia S., Carbrello C., Bell D., Zydney A.L. (2019). Impact of protein fouling on nanoparticle capture within the Viresolve® Pro and Viresolve® NFP virus removal membranes. Biotechnol. Bioeng..

[12] Pageni P., Yang P., Bam M., Zhu T., Chen Y.P., Decho A.W., Nagarkatti M., Tang C. (2018). Recyclable magnetic nanoparticles grafted with antimicrobial metallopolymer-antibiotic bioconjugates. Biomaterials.

[13] Singh P., Gupta R., Choudhary M., Pinnaka A.K., Kumar R., Bhalla V. (2018). Drug and nanoparticle mediated rapid naked eye water test for pathogens detection. Sens. Actuators B.

[14] Yang J., Wang K., Xu H., Yan W., Jin Q., Cui D. (2019). Detection platforms for point-of-care testing based on colorimetric, luminescent and magnetic assays: A review. Talanta.

[15] Guo T., Wang C., Zhou H., Zhang Y., Ma L. (2021). A multifunctional near-infrared fluorescent sensing material based on core-shell upconversion nanoparticles@magnetic nanoparticles and molecularly imprinted polymers for detection of deltamethrin. Microchim. Acta.

[16] Hakeem D.A., Su S., Mo Z., Wen H. (2022). Upconversion luminescent nanomaterials: A promising new platform for food safety analysis. Crit. Rev. Food Sci. Nutr..

[17] Hagan A.K., Zuchner T. (2011). Lanthanide-based time-resolved luminescence immunoassays. Anal. Bioanal. Chem..

[18] Päkkilä H., Malmi E., Lahtinen S., Soukka T. (2014). Rapid homogeneous immunoassay for cardiac troponin I using switchable lanthanide luminescence. Biosens. Bioelectron..

[19] Lim J., Lanni C., Evarts E.R., Lanni F., Tilton R.D., Majetich S.A. (2011). Magnetophoresis of Nanoparticles. ACS Nano.

[20] Suwa M., Watarai H. (2011). Magnetoanalysis of micro/nanoparticles: A review. Anal. Chim. Acta.

[21] Sun X., Lei C., Guo L., Zhou Y. (2016). Separable detecting of Escherichia coli O157H:H7 by a giant magneto-resistance-based bio-sensing system. Sens. Actuators B.

[22] Wang S.X., Li G. (2008). Advances in giant magnetoresistance biosensors with magnetic nanoparticle tags: Review and outlook. IEEE Trans. Magn..

[23] Nikitin M.P., Orlov A.V., Znoyko S.L., Bragina V.A., Gorshkov B.G., Ksenevich T.I., Cherkasov V.R., Nikitin P.I. (2018). Multiplex biosensing with highly sensitive magnetic nanoparticle quantification method. J. Magn. Magn. Mater..

[24] Dey C., Yari P., Wu K. (2023). Recent advances in magnetoresistance biosensors: A short review. Nano Futures.

[25] Ren C., Bayin Q., Feng S., Fu Y., Ma X., Guo J. (2020). Biomarkers detection with magnetoresistance-based sensors. Biosens. Bioelectron..

[26] Quynh L.K., Tu B.D., Dang D.X., Viet D.Q., Hien L.T., Huong Giang D.T., Duc N.H. (2016). Detection of magnetic nanoparticles using simple AMR sensors in Wheatstone bridge. J. Sci. Adv. Mater. Devices.

[27] Jin Z., Koo T.M., Kim M.S., Al-Mahdawi M., Oogane M., Ando Y., Kim Y.K. (2021). Highly-sensitive magnetic sensor for detecting magnetic nanoparticles based on magnetic tunnel junctions at a low static field. AIP Adv..

[28] Chung K.H., Kim S.N., Lim S.H. (2018). Magnetic parameters in giant magnetoresistance spin valve and their roles in magnetoresistance sensitivity. Thin Solid Film..

[29] Schotter J., Kamp P.B., Becker A., Pühler A., Reiss G., Brückl H. (2004). Comparison of a prototype magnetoresistive biosensor to standard fluorescent DNA detection. Biosens. Bioelectron..

[30] Choi J., Gani A.W., Bechstein D.J.B., Lee J.-R., Utz P.J., Wang S.X. (2016). Portable, one-step, and rapid GMR biosensor platform with smartphone interface. Biosens. Bioelectron..

[31] Sushruth M., Ding J., Duczynski J., Woodward R.C., Begley R.A., Fangohr H., Fuller R.O., Adeyeye A.O., Kostylev M., Metaxas P. (2016). Resonance-based detection of magnetic nanoparticles and microbeads using nanopatterned ferromagnets. Phys. Rev. Appl..

[32] Kittel C. (1948). On the theory of ferromagnetic resonance absorption. Phys. Rev..

[33] Stancil D.D., Prabhakar A. (2009). Spin Waves Theory and Applications.

[34] Chatterjee E., Marr T., Dhagat P., Remcho V.T. (2011). A microfluidic sensor based on ferromagnetic resonance induced in magnetic bead labels. Sens. Actuators B.

[35] Manzin A., Ferroro R., Vicentini M. (2022). Application of magnonic crystals in magnetic bead detection. Nanomaterials.

[36] Banholzer A., Narkowicz R., Hasssel A., Meckenstock R., Stienen S., Posth O., Suter D., Farle M., Lindner J. (2011). Visualization of spin dynamics in single nanosized magnetic elements. Nanotechnology.

[37] Cansever H., Anwar M.S., Stienen S., Lenz K., Narkowicz R., Hlawacek G., Potzger K., Hellwig O., Fassbender J., Lindner J. (2022). Resonance behavior of embedded and freestanding microscale ferromagnets. Sci. Rep..

[38] Cardoso S., Leitao D.C., Dias T.M., Valadeiro J., Silva M.D., Chicharo A., Silverio V., Gaspar J., Freitas P.P. (2017). Challenges and trends in magnetic sensor integration with microfluidics for biomedical applications. J. Phys. D Appl. Phys..

[39] Giouroudi I., Kokkinis G. (2017). Recent advances in magnetic microfluidic biosensors. Nanomaterials.

[40] DIXIT C.K., Kaushik A. (2016). Microfluidics for Biologists Fundamentals and Applications.

[41] Dowling R., Kostylev M. (2023). External magnetic fields enhance capture of magnetic nanoparticles flowing through molded microfluidic channel by ferromagnetic nanostructures. arXiv.

[42] Boero G., Bouterfas M., Massin C., Vincent F., Besse P.-A., Popovic R.S., Schweiger A. (2003). Electron-spin resonance probe based on a 100 μm planar microcoil. Rev. Sci. Instrum..

[43] Narkowicz R., Suter D., Stonies R. (2005). Planar microresonators for EPR experiments. J. Magn. Reson..

[44] McDonald J.C., Duffy D.C., Anderson J.R., Chiu D.T., Wu H., Schueller O.J.A., Whitesides G.M. (2000). Fabrication of microfluidic systems in poly(dimethylsiloxane). Electrophoresis.

[45] Becker H., Gӓrtner C. (2000). Polymer microfabrication methods for microfluidic analytical applications. Electrophoresis.

[46] Fiorini G.S., Chiu D.T. (2005). Disposable microfluidic devices: Fabrication, function, and application. BioTechniques.

[47] Stone H.A., Stroock A.D., Ajdari A. (2004). Engineering flows in small devices: Microfluidics toward a lab-on-a-chip. Annu. Rev. Fluid Mech..

[48] Ren K., Zhou J., Wu H. (2013). Materials for microfluidic chip fabrication. Acc. Chem. Res..

[49] Felton H., Hughes R., Diaz-Gaxiola A. (2021). Negligible-cost microfluidic device fabrication using 3D-printed interconnecting channel scaffolds. PLoS ONE.

[50] Montoya E., McKinnon T., Zamani A., Girt E., Heinrich B. (2014). Broadband ferromagnetic resonance system and methods for ultrathin magnetic films. J. Magn. Magn. Mater..

[51] Maksymov I.S., Mikhail K. (2015). Broadband stripline ferromagnetic resonance spectroscopy of ferromagnetic films, multilayers and nanostructures. Phys. E Low Dimens. Syst. Nanostructures.

[52] Ivanov E.N., Kostylev M. (2014). Extremely high-resolution measurements of microwave magnetisation dynamics in magnetic thin films and nanostructures. arXiv.

[53] Vansteenkiste A., Leliaert J., Dvornik M., Helsen M., Garcia-Sanchez F., Waeyenberge B.V. (2014). The design and verification of MuMax3. AIP Adv..

[54] Vansteenkiste A., Van de Wiele B. (2011). MuMax: A new high-performance micromagnetic simulation tool. J. Magn. Magn. Mater..

[55] Berthier J., Brakke K.A. (2012). The Physics of Microdroplets.

[56] Tripathi D., Tripathi S., Rawat R.K., Chauhan P. (2023). Highly sensitive humidity sensor based on freestanding graphene oxide sheets for respiration and moisture detection. J. Electron. Mater..

[57] Krivoruchko V.N., Marchenko A.I. (2011). Apparent sixfold configurational anisotropy and spatial confinement of ferromagnetic resonances in hexagonal magnetic antidot lattices. J. Appl. Phys..

[58] Neusser S., Botters B., Grundler D. (2008). Localization, confinement, and field-controlled propagation of spin waves in Ni_80_Fe_20_ antidot lattices. Phys. Rev. B.

[59] Dowling R., Kostylev M. (2023). Enhancing the capture of magnetic nanoparticles inside of ferromagnetic nanostructures using external magnetic fields. arXiv.

